# Induction of anti-EGFR immune response with mimotopes identified from a phage display peptide library by panitumumab

**DOI:** 10.18632/oncotarget.12167

**Published:** 2016-09-21

**Authors:** Aidong Wang, Ming Cui, Hong Qu, Jiabo Di, Zaozao Wang, Jiadi Xing, Fan Wu, Wei Wu, Xicheng Wang, Lin Shen, Beihai Jiang, Xiangqian Su

**Affiliations:** ^1^ Key laboratory of Carcinogenesis and Translational Research (Ministry of Education), Department of Gastrointestinal Surgery IV, Peking University Cancer Hospital & Institute, Beijing 100142, China; ^2^ Center for Bioinformatics, State Key Laboratory of Protein and Plant Gene Research, College of Life Sciences, Peking University, Beijing, 100871, China; ^3^ Key laboratory of Carcinogenesis and Translational Research (Ministry of Education), Department of Gastrointestinal Oncology, Peking University Cancer Hospital & Institute, Beijing 100142, China

**Keywords:** EGFR, panitumumab, mimotope, peptide vaccine

## Abstract

The epidermal growth factor receptor (EGFR) is overexpressed in several epithelial tumors. Anti-EGFR humanized monoclonal antibodies, cetuximab and panitumumab, in combination with chemotherapy have improved the prognosis for patients with wild-type RAS tumors. To identify mimotopes of EGFR and develop mimotope-based EGFR vaccines, we screened a phage display peptide library with panitumumab. Two EGFR mimotopes P19 and P26, which could be recognized by panitumumab specifically, were isolated. To enhance the immune responses, we generated recombinant proteins of P19 or P26 fused to a heat-shock cognate protein 70 (Hsc70), and evaluated the efficacy of Hsc70-P19 and Hsc70-P26 as vaccines *in vivo*. Immunization with Hsc70-P19 or Hsc70-P26 fusion protein stimulated the immune system to produce specific antibodies against peptides as well as EGFR. Moreover, antibodies elicited against mimotopes could induce antibody-dependent cellular cytotoxicity (ADCC), complement-dependent cytotoxicity (CDC), and inhibit the proliferation of EGFR-overexpressing A431 cells. Treatment with Hsc70-P19 and Hsc70-P26 significantly reduced tumor growth in BALB/c transplantable lung cancer models. Although there was no sequence homology between the phage-derived peptides and EGFR by alignments, both peptides mimic the conformational structure of EGFR binding to panitumumab. In conclusion, the mimotopes we identified from phage display peptide library could be promising candidate vaccines for active anti-EGFR immunotherapy against cancers.

## INTRODUCTION

The epidermal growth factor receptor (EGFR), a transmembrane receptor tyrosine kinase, is overexpressed in a variety of cancers, and associated with tumor progression and poor prognosis [1]. It promotes cell proliferation, migration, differentiation, and survival by activating MAPK and PI3K signaling pathways [1]. As a critical therapeutic target in cancers, therapeutic agents targeting EGFR consist of both EGFR-tyrosine kinase inhibitors and monoclonal antibodies [1–3]. Kinase inhibitors, including gefitinib and erlotinib, have been proven effective in a number of patients with non-small cell lung cancers containing constitutively active EGFR mutations [2]. On the other hand, humanized monoclonal antibodies, cetuximab and panitumumab, appear to improve survival in patients with wild-type KRAS metastatic colorectal cancer [3].

However, there are many limitations of current FDA-approved monoclonal antibody therapies, including short half-life, the need to repeat administration, inadequate tissue distribution, possible immunogenicity against therapeutic antibodies, the development of resistance, and high cost [4–6]. Alternatively, to overcome these disadvantages, peptide-based vaccines are able to induce active immunity against tumor-specific antigens, develop immunological memory, and provide possible methods to circumvent resistance [4–8]. Among them, Rindopepimut, the EGFR-derived 14-mer peptide vaccine consisting of the mutation site of EGFRvIII, has been demonstrated as a safe and potentially effective drug for the treatment of glioblastoma multiforme (GBM) in phase II clinical trials [9, 10].

In addition to the peptide vaccines derived from the amino acid sequence of antigens, peptide mimotopes, which are composed of amino acids different from those of native antigens, can mimic antigen-antibody binding sites structurally [5–7]. Active immunization with mimotopes would elicit antibodies against the corresponding epitopes. Since mimotopes can be identified from phage display peptide libraries [5–7], here we screened a phage display 12-mer peptide library, and identified two peptides, P19 and P26, that mimic the EGFR epitopes which can be recognized by panitumumab. The results showed that P19 and P26 contributed to the comformational epitopes of EGFR. Both of them could induce antibody responses to EGFR as well as cellular immune responses in mice. Taken together, peptides P19 and P26 are potential vaccine candidates for the treatment of cancers with EGFR overexpression.

## RESULTS

### Identification of phages binding to panitumumab

A 12-mer phage display peptide library was screened on panitumumab to isolate the epitope-mimicking peptides. After three rounds of biopanning, selectively binding phages were enriched. The phage titer increased from 1.2×10^5^ pfu in the first round to 2.8×10^8^ pfu in the third round.

**Figure 1:**
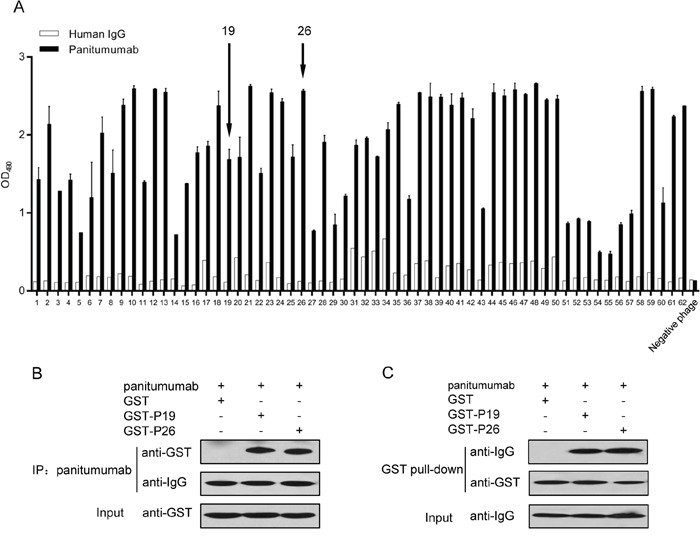
Selection of mimotopes from phage display peptide library on panitumumab **A.** Single phage clones bind specifically to panitumumab but not to control human IgG. Phage binding was detected by ELISA. Data are presented as the mean OD values (±SDs) of 3 independent experiments performed in triplicate. **B.** Co-immunoprecipitation (co-IP) to validate panitumumab interaction with GST-P19 and GST-P26, but not with GST. **C.** GST pull-down assay to confirm the binding of GST-P19 and GST-P26 to panitumumab. Purified GST fusion proteins were coincubated with panitumumab. The complexes were precipitated as described, followed by Western blot with antibodies against GST and human IgG, respectively.

Subsequently, the specificity of phage clones binding to panitumumab was determined by ELISA. After three rounds of biopanning, 17.4% (62/356) of the phage clones analyzed showed binding activity to panitumumab (Figure 1A). We sequenced 50 positive phage clones, among which P26 (VPGWSQAFMALA) and P19 (DTDWVRMRDSAR) were the most frequent peptide sequences, suggesting that these two peptide sequences may define common peptides recognized by panitumumab. Accordingly, two distinct consensus motifs HVPG(Q)W(H)SQ and DTX_n_RD were identified (Table 1). Therefore, P19 and P26 were chosen for further studies.

**Table 1:** Peptide sequences selected from phage display peptide library screening on panitumumab

	Amino acid sequence of phage clone														Frequency
Motif I															
			V	P	G	W	S	Q	A	F	M	A	L	A	21/50 (42%)
	N	H	V	P	Q	H	T	P	I	H	L	R			2/50 (4%)
	A	H	V	P	Q	H	H	A	M	T	G	R			2/50 (4%)
			V	P	G	W	S	Q	T	F	M	P	L	A	1/50 (2%)
	G	H	V	T	Q	H	S	Q	R	G	Y	S			1/50 (2%)
	G	H	V	P	Q	H	A	R	L	L	G	L			1/50 (2%)
	T	H	V	P	E	H	L	L	K	P	R	P			1/50 (2%)
	G	H	V	Q	Q	H	D	V	H	S	I	R			1/50 (2%)
	Y	H	V	P	E	H	A	V	R	L	G	P			1/50 (2%)
	K	H	V	L	Q	H	Q	T	S	M	T	M			1/50 (2%)
	K	H	V	V	Q	H	E	Y	A	P	N	A			1/50 (2%)
	H	H	T	D	E	H	W	L	F	A	K	K			1/50 (2%)
	L	S	H	R	T	H	D	R	I	G	F	T			1/50 (2%)
		Y	P	P	Q	E	R	T	V	H	K	Y	V		1/50 (2%)
Consensus motif I		H	V	P	G/Q	W/H	S	Q							
Motif II															
			D	T	D	W	V	R	M	R	D	S	A	R	9/50 (18%)
			D	M	T	Y	M	E	R	R	D	S	A	R	2/50 (4%)
			D	A	Y	T	Q	L	R	D	R	M	R	Q	1/50 (2%)
		I	D	G	Y	P	G	S	P	R	L	P	W		1/50 (2%)
			W	H	W	T	W	P	A	D	V	G	W	V	1/50 (2%)
Consensus motif II			D	T	X	X	X	X	X	R	D				

### P19 and P26 specifically bind to panitumumab

To verify the binding of P19 and P26 to panitumumab, we performed co-IP and GST pull-down assay. For these experiments, oligonucleotide encoding the peptide was expressed as the C-terminal extension of GST (Supplementary Figure 1A and 1B). Co-IP assay showed that panitumumab could precipitate GST-P19 and GST-P26, but not GST (Figure 1B). The GST pull-down assay further confirmed the binding of GST-P19 and GST-P26 to panitumumab, whereas GST failed to precipitate panitumumab (Figure 1C). These results suggested that P19 and P26 specifically bind to panitumumab.

### P19 and P26 inhibit the binding of panitumumab to EGFR

**Figure 2:**
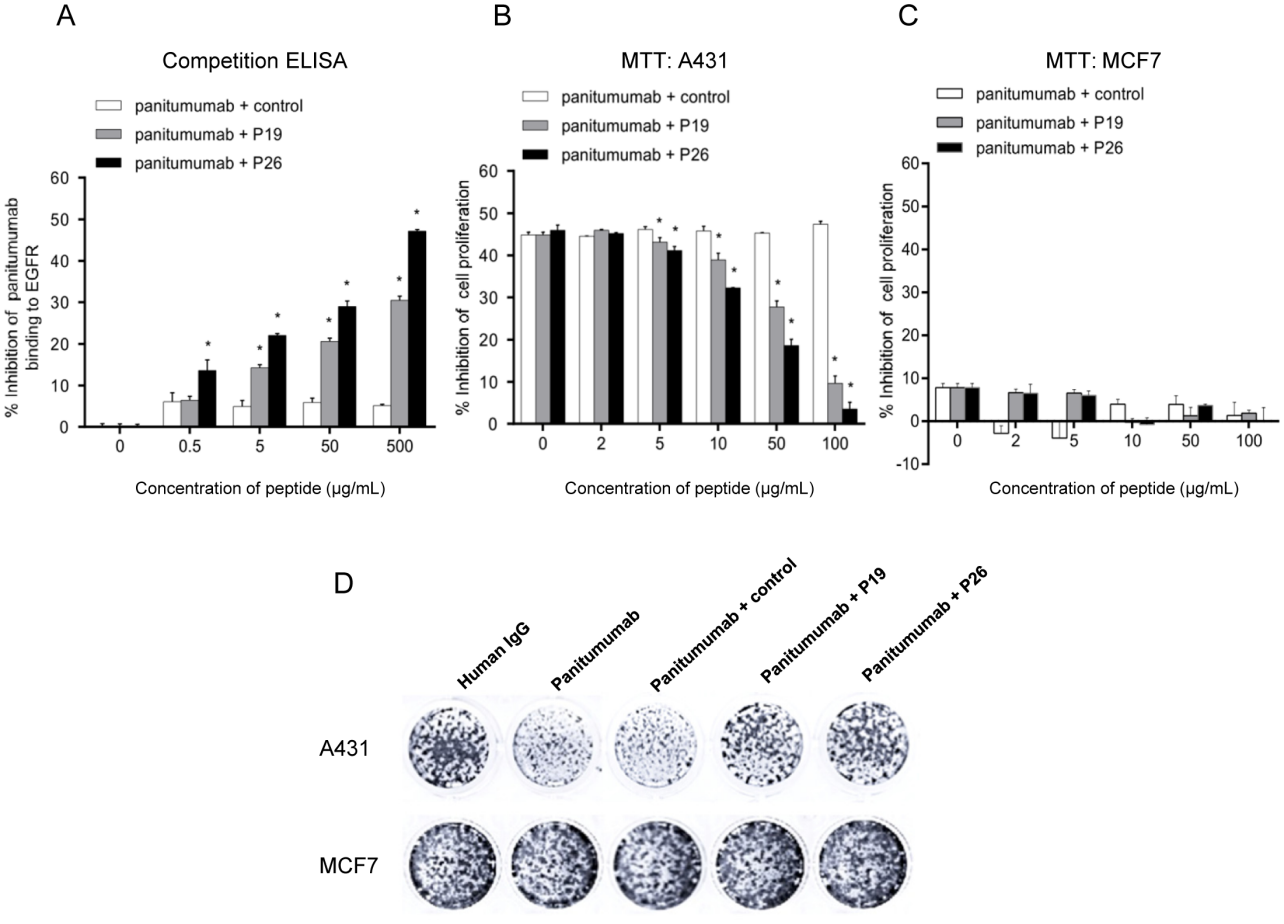
Peptides P19 and P26 inhibited the function of panitumumab **A.** ELISA assays demonstrated P19 and P26 inhibited the binding of panitumumab to EGFR. Percentage of inhibition was calculated as follows: (OD_panitumumab_ - OD_panitumumab with peptide_) ×100%/OD_panitumumab_. **B.** MTT assay suggested that P19 and P26 blocked the effect of panitumumb on inhibition of cell proliferation in A431 cells, but not in MCF7 cells **C.** Percentage of inhibition was calculated as follows: (OD_human IgG_ - OD_panitumumab with peptide_)×100%/OD_human IgG_. Data are shown as the mean OD values (±SDs) of 3 independent experiments performed in triplicate. Statistically significant differences are indicated. *, *P* < 0.05. **D.** Colony formation assay of A431 and MCF7 cells cultured in the presence of panitumumab with or without peptides.

The ability of the peptides to inhibit the binding of the panitumumab to EGFR was determined by ELISA. When panitumumab was allowed to bind to EGFR in the presence of increasing concentrations of P19 and P26, a dose-dependent inhibition of binding up to 30.48% and 47.15% was found for peptides P19 and P26, respectively (Figure 2A). However, binding of panitumumab to EGFR could not be significantly inhibited by the control peptide (Figure 2A). These results suggested that P19 and P26 are the mimotopes of EGFR.

### P19 and P26 prevent panitumumab from inhibiting tumor cell growth

It has been well demonstrated that panitumumab could bind to EGFR, and inhibit the growth of EGFR-overexpressing cell lines [11]. Since our results showed that P19 and P26 not only bind to panitumumab, but also attenuate the binding of panitumumab to EGFR, we investigated whether P19 and P26 could prevent panitumumab from inhibiting tumor cell growth. Firstly, we verified the levels of EGFR in A431 [11] and MCF7 cells [12], which expressed high and low levels of EGFR, respectively (Supplementary Figure 2A and 2B). Secondly, we confirmed the function of panitumumab in inhibiting proliferation of A431, but not MCF7 cells (Supplementary Figure 2C). Thirdly, we demonstrated that P19 and P26 alone had no significant effects on the proliferation of A431 or MCF7 cells (Supplementary Figure 2D). Accordingly, by preincubating panitumumab with peptides, the response of A431 cells to panitumumab was significantly blocked by P19 and P26 in a dose-dependent manner (Figure 2B). However, similar concentrations of control peptide had no significant effects on the inhibition by panitumumab (Figure 2B). Moreover, neither of the peptide combined with panitumumab had growth inhibition of MCF7 cells (Figure 2C). Colony formation assays also showed similar trends of effects of peptide P19 and P26 (Figure 2D). These results demonstrated that P19 and P26 are EGFR mimotopes, and prevent panitumumab from inhibiting tumor cell growth.

### Immune responses induced by Hsc70-P19 and Hsc70-P26

**Figure 3:**
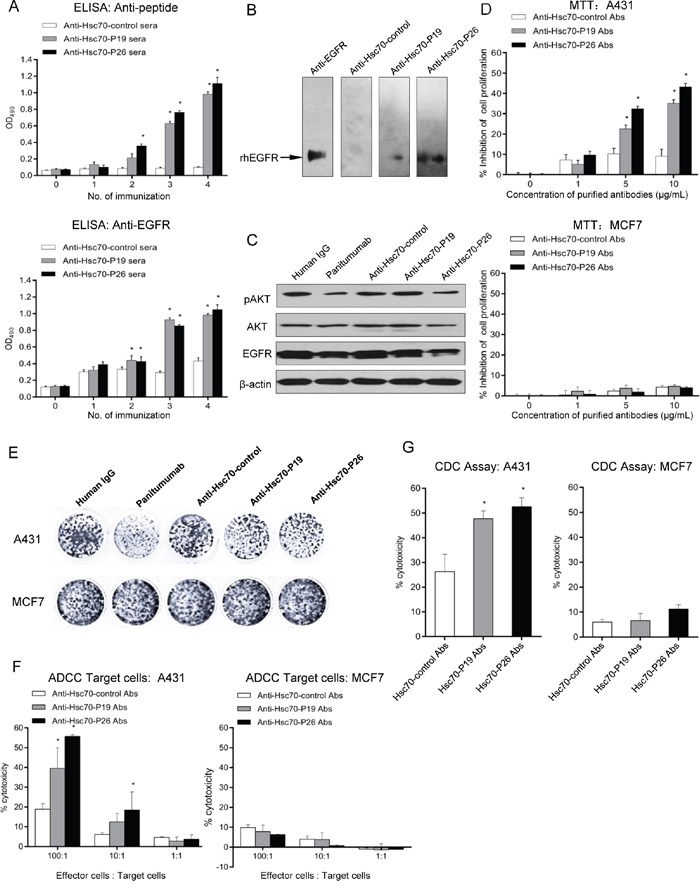
Antibody responses induced by mimotope immunization **A.** ELISA was performed to determine the anti-peptide (upper panel) and anti-EGFR (lower panel) antibody responses with GST fusion protein and rhEGFR-coated plates, respectively. Mice sera were diluted at 1:2000 and 1:100, respectively. **B.** Western blot analysis of anti-EGFR antibody from immunized mice. **C.** Western blot to determine the role of mimotope antibodies on EGFR-mediated signaling. **D.** MTT assays of A431 (upper panel) and MCF7 cells (lower panel) treated with purified mimotope antibodies. **E.** Colony formation assay of A431 and MCF7 cells treated with purified mimotope antibodies. **F.** ADCC activity of anti-mimotope antibodies. Human PBMC were used as effector cells. The effector to target A431 (left panel) or MCF7 cells (right panel) (E:T) ratio was 1:1, 10:1, and 100:1. Cytotoxicity was calculated by the formula: (experimental - effector spontaneous - target spontaneous) ×100%/(target maximum - target spontaneous). **G.** CDC activity of mice immunized with mimotopes. A431 (left panel) or MCF7 cells (right panel) were cultured with purified mimotope antibodies and human sera. The cytotoxicity was calculated. Data are shown as the mean (±SDs) of 3 independent experiments performed in triplicate. Statistically significant differences are indicated. *, *P* < 0.05.

Peptide P19 and P26 were expressed as Hsc70 peptide fusion proteins, Hsc70-P19 and Hsc70-P26, respectively (Supplementary Figure 3A, 3B and 3C). The immune responses induced by Hsc70 fusion proteins in BALB/c mice were examined by ELISA. Mice immunized with Hsc70-P19 and Hsc70-P26 generated an antibody response against P19 or P26, respectively (Figure 3A upper panel), as well as against rhEGFR (Figure 3A lower panel). The anti-EGFR response was lower than the anti-P19 or anti-P26 response. The anti-P19 and anti-P26 responses were detectable after the second immunization (Figure 3A upper pannel), whereas the anti-EGFR responses could be observed after the third immunization (Figure 3A lower pannel). There was no significant anti-peptide or anti-EGFR responses in mice immunized with Hsc70-control. Moreover, the specificity of antibodies induced by Hsc70-p19 or Hsc70-p26 in mice was confirmed by western blot. Although no response was detected with anti-Hsc70-control serum, anti-Hsc70-P19 and anti-Hsc70-P26 serum specifically bound to rhEGFR, as commercial anti-EGFR antibody did. The response was stronger in anti-Hsc70-P26 serum than in anti-Hsc70-P19 serum (Figure 3B).

### Anti-peptide antibodies inhibited receptor-mediated signaling

To investigate the mechanism underlying antibody-mediated inhibition of A431 cell growth, we assessed the effects of anti-Hsc70-P19 and anti-Hsc70-P26 antibodies on receptor-mediated signaling. Consistent with panitumumab treatment, cell exposed to anti-Hsc70-P26 antibody showed a significant reduction in pAKT and EGFR, compared with anti-Hsc70-control antibody therapy. However, treatment of anti-Hsc70-P19 antibody produced no significant changes in pAKT, AKT, or EGFR (Figure 3C).

### Anti-proliferation abilities of peptide antibodies on EGFR-overexpressing cells

Since antibodies elicited by Hsc70-P19 and Hsc70-P26 were able to bind to EGFR, we assessed the growth inhibiting effects of these antibodies on tumor cells expressing high levels of EGFR by MTT and colony formation assays. The results of MTT assays suggested that antibodies induced by Hsc70-P19 and Hsc70-P26 significantly inhibited the growth of A431 (Figure 3D upper panel) and SW480 cells (Supplementary Figure 4) in a dose-dependent manner. However, antibodies against Hsc70-control had minimal effects on the proliferation of A431 and SW480 cells (Figure 3D upper panel and Supplementary Figure 4). For MCF7 cells with low levels of EGFR, neither of the antibodies could significantly prevent cell proliferation (Figure 3D lower panel). Moreover, the results of colony formation assays showed that similar trends of proliferation inhibition by antibodies induced by Hsc70-P19 and Hsc70-P26 were found in A431 cells, compared with MCF7 cells (Figure 3E). These results indicated that antibodies against P19 and P26 inhibit the growth of EGFR-overexpressing tumor cells.

### Antibodies induced by Hsc70-P19 and Hsc70-P26 mediated ADCC and CDC

Antibodies purified from immunized mice were evaluated for their ability to mediate tumor cell lysis in an ADCC-dependent manner. Incubation of the A431 cells with the purified antibodies from the mice immunized with Hsc70-P19 and Hsc70-P26 elicited 39.6% and 55.7% cell lysis, respectively, at E:T ratio of 100:1. However, antibodies obtained from Hsc70-control immunized mice mediated A431 cell lysis by only 21.9%. Thus, the ADCC-mediated tumor cell lysis was significantly higher in Hsc70-P19 and Hsc70-P26-immunized groups, compared with Hsc70-control-immunized group (Figure 3F left panel). Moreover, antibodies elicited in immunized mice could not invoke lysis of EGFR low-expressing MCF7 cells (Figure 3F, right panel). The ability of immunized antibodies to mediate CDC was also analyzed. The results revealed that 10μg/mL of anti-Hsc70-P19 or anti-Hsc70-P26 antibodies mediated notable CDC to kill A431 cells by co-incubating with human serum, compared with anti-Hsc70-control antibodies (Figure 3G left panel). However, there was no obvious antibody-mediated CDC to kill MCF7 cells (Figure 3G, right panel).

### Development of cellular immune responses

**Figure 4:**
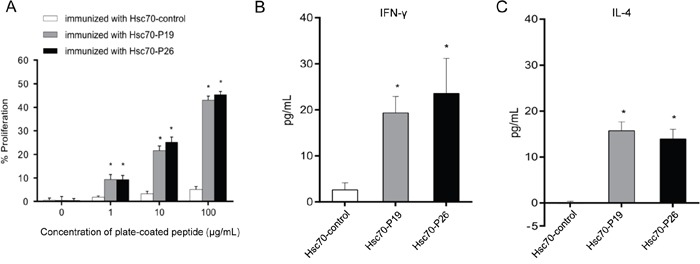
Induction of cellular immune responses by mimotope immunization **A.** Determination of splenocyte proliferation in mice immunized with mimotopes. The splenocytes from immunized mice were cultured in 96-well plates coated with P19, P26 or control peptide. **B.** ELISA to detect cytokine IFN-γ (left panel) and IL-4 (right panel) in sera of immunized mice. Data are presented as the mean (±SDs) of 3 independent experiments performed in triplicate. Statistically significant differences are indicated. *, *P* < 0.05.

Cellular immunity elicited by Hsc70-P19, Hsc70-P26, and Hsc70-control immunization was determined by both splenocyte proliferation assay and cytokine assay. The splenocytes of five mice were isolated, and stimulated with P19 and P26 *in vitro*. The results suggested that splenocyte proliferation was induced in mice immunized with Hsc70-P19 or Hsc70-P26, but not in mice immunized with Hsc70-control (Figure 4A). Moreover, after two weeks of the third immunization, Hsc70-P19 and Hsc70-P26 groups developed significantly higher levels of IFN-γ and IL-4 compared to the Hsc70-control group (Figure 4B and 4C).

### Treatment with Hsc70 peptide fusion protein prevents tumor growth *in vivo*

**Figure 5:**
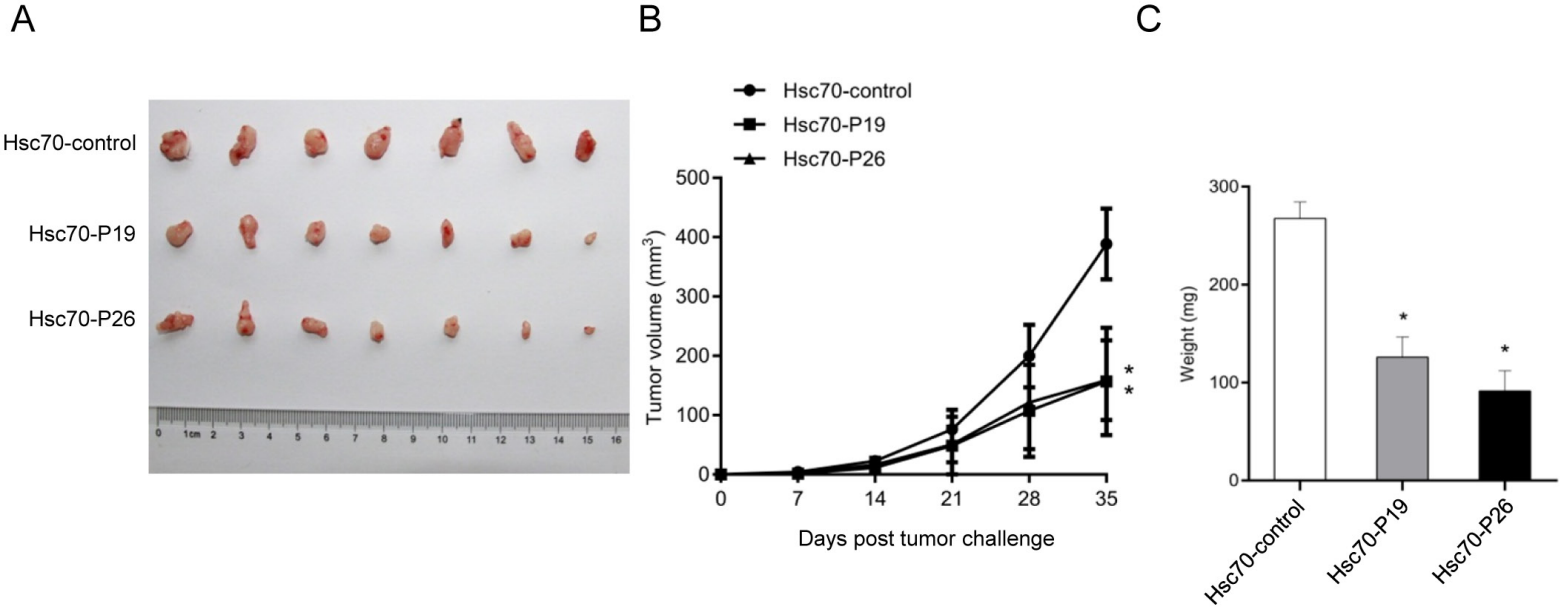
Effects of treatment with Hsc70-peptide fusion protein in transplantable lung tumor model of mouse **A.** Photograph showed tumor formation in BALB/c SCID mice injected with Hsc70-P19, Hsc70-P26, and Hsc70-control. **B.** Hsc70-P19 and Hsc70-P26 treatment elicited a significant delay in tumor growth, compared with Hsc70-control treatment. **C.** Tumor weight was significantly inhibited in mice with Hsc70-P19 and Hsc70-P26 treatment, compared with Hsc70-control treatment. Data are shown as the mean (±SDs). Statistically significant differences are indicated. *, *P* < 0.05.

To evaluate the inhibitory effects of Hsc70 fusion protein *in vivo*, we used a lung cancer transplantable model of BALB/c SCID mice [4]. The results suggested that Hsc70-P19 and Hsc70-P26 significantly inhibited tumor growth and development, compared with Hsc70-control therapy (Figure 5A and 5B). Accordingly, tumor weight in Hsc70-P19 and Hsc70-P26 group was significantly lower than that in Hsc70-control group (Figure 5C).

### P19 and P26 mimic the interaction of EGFR with panitumumab

To further confirm that P19 and P26 were EGFR mimotopes for binding to panitumumab, we compared the interface structure of peptide-panitumumab complexes with EGFR-panitumumab complex.

Firstly, the 3D models for panitumumab Fab was built. Since no crystal structure of panitumumab was available in the database, we generated a structure model of the variable regions in the heavy and light chains of panitumumab using SWISS-MODEL server [13–15]. After the alignment of the target and the template sequences, the crystal structure of the heavy chain of the germline antibody PGT121-GL Fab (PDB: 4fqq) and the light chain of the RSV-neutralizing human antibody D25 (PDB: 4jha) were selected as heavy and light chain templates for model building, respectively. Accordingly, the SWISS-MODEL provided the 3D models for the variable regions of the heavy and light chains of panitumumab. The 3D model for panitumumab Fab was generated by docking the two variable regions together using the docking program in Insight II software.

**Figure 6:**
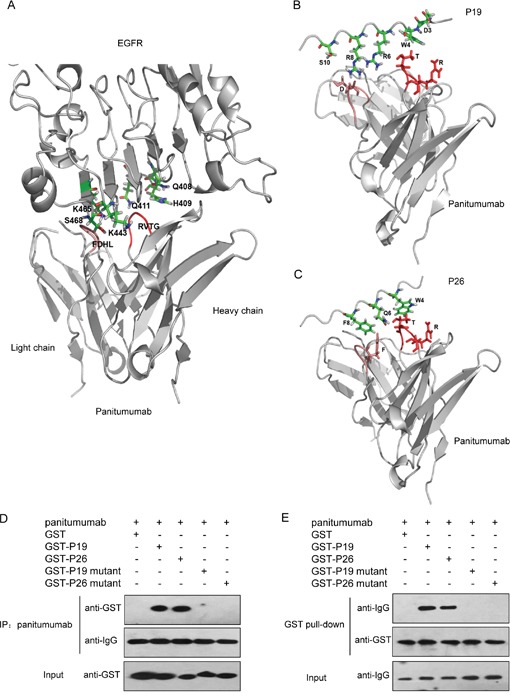
Three-dimensional models of the EGFR-panitumumab and mimotope-panitumumab complex and determination of functional amino acides in peptides **A.** A side view of the interaction of EGFR domain III with panitumumab Fab. The backbone of EGFR and panitumumab Fab are shown in gray. CDR3 of V_H_ and V_L_ contributing to the crucial interaction with EGFR are shown in red and light red, respectively. Amino acids from EGFR that are directly involved in binding to panitumumab Fab are displayed. B. Detailed view of the P19-panitumumab interface. **C.** Detailed view of the P26-panitumumab complex. The backbone of panitumumab Fab is shown in gray. Amino acids in CDR3 of V_H_ and V_L_ which contributed to the interaction with P19 and P26 are shown in red and light red, respectively. Amino acids of P19 or P26 involved in the interaction with panitumumab Fab are shown. **D.** co-IP to evaluate panitumumab interaction with GST-P19 mutant and GST-P26 mutant. **E.** GST pull-down assay to determine the binding of GST-P19 mutant and GST-P26 mutant to panitumumab. Purified GST fusion proteins were coincubated with panitumumab. The complexes were precipitated as described, followed by western blot with antibodies against GST and human IgG, respectively.

Secondly, the interaction of EGFR with panitumumab was analyzed. According to the crystal structure of human EGFR [16] and the predicted model of panitumumab Fab, the EGFR-panitumumab complex was modeled using the HEX 8.0.0 program [17]. The panitumumab Fab binds to EGFR domain III. Moreover, the complementarity determining regions (CDRs) 3 in variable regions of both heavy and light chains (V_L_ and V_H_, respectively) of the Fab contributed to the majority of the interactions with EGFR (Figure 6A, in red and light red, respectively), with additional contributions from CDR2 in V_H_.

For CDR3 in V_H_, amino acids 78-81 (RVTG) participated in the interaction with Q408[EGFR] and H409[EGFR] in EGFR domain III. Among them, T80[V_H_] made hydrogen bond with Q411[EGFR]. In addition, for CDR2 in V_H_, amino acids 31-36 (YYSGNT) was anchored over the surface of domain III by forming hydrogen bonds (Y31[V_H_] to Q408[EGFR], S33[V_H_] to Q384[EGFR] or R353[EGFR], N35[V_H_] to V417[EGFR], and T36[V_H_] to S418[EGFR]). Moreover, amino acids 72-75 (FDHL) in CDR3 of V_L_ could form hydrogen bonds and electrostatic interaction with I467[EGFR], S468[EGFR], K465[EGFR], and K443[EGFR], and contribute to the interaction with domain III (Figure 6A). Thirdly, to demonstrate whether the functional amino acids in P19 or P26 partially overlap with the residues of EGFR domain III, we compared the interfaces formed by panitumumab with EGFR and peptides, respectively. Since there was no sequence homology between P19 or P26 and EGFR, the docking program in Insight II software was used to model the complexes of P19 or P26 with panitumumab.

In the P19-panitumumab complex model, as shown in Figure 6B, R78[V_H_] and T80[V_H_] in RVTG[V_H_] could interact with D3[P19] and W4[P19], which occupied approximately the same positions of Q408[EGFR] and H409[EGFR], respectively, on the surface of EGFR domain III. Moreover, D73[V_L_] in FDLH[V_L_] could make electrostatic interactions with R6[P19] and R8[P19], which were similar to K443[EGFR] and K465[EGFR], respectively. In addition, D73[V_L_] and H75[V_L_] could form hydrogen bonds with S10[P19], which harbored the same position as S468[EGFR].

In the P26-panitumumab complex model, as shown in Figure 6C, R78[V_H_] and T80[V_H_] in RVTG[V_H_] could interact with W4[P26] and Q6[P26], which were well represented by Q408[EGFR] and H409[EGFR], respectively. Moreover, the π-π interaction between F72[V_L_] in FDLH[V_L_] and F8[P26] was identified, as well as the hydrophobic interactions between D73[V_L_] or H[V_L_] in FDLH[V_L_] and A10[P26], L11[P26] or A12[P26], which enhanced the binding of P26 to panitumumab. Taken together, the modeling structures raised the possibility that P19 and P26 could mimic the epitope of EGFR binding to panitumumab.

To verify the contribution of functional amino acids in P19 and P26 binding to panitumumab, we generated GST-P19 mutant (DTAAVAMADAAR) and GST-P26 mutant (VPGASAAFMAAA) (Supplementary Table 1), and then performed co-IP and GST pull-down assay. The results showed that none of these mutants could interact with panitumumab anymore (Figure 6D and 6E), suggesting that the crucial residues predicted by the modeling structures are bona fide functional amino acids in peptide-EGFR interaction.

## DISCUSSION

The anti-EGFR monoclonal antibodies cetuximab and panitumumab are administered to treat colorectal cancer patients with wild-type RAS tumors, and demonstrated to improve the survival of patients. However, a large number of patients did not respond to this treatment, or even developed resistance [18, 19]. In addition, several problems are associated with antibodies treatment, including high cost, short half-life, and adverse events developed from toxicity [4–6]. Active immunization using peptide vaccines can induce more durable antibodies to recognize specific antigens, and develop immunological memory, which is critical under the circumstance of disease recurrence [4–8].

In this study, two EGFR epitopes mimics (P19 and P26) were isolated from the phage display peptide library by panitumumab. And these mimotope vaccines could induce antibodies against EGFR, which have the similar effects as panitumumab. Although there was no sequence homology between the mimotopes and EGFR according to sequence alignment, 3D modeling suggests that P19 and P26 resemble the conformational structure of EGFR surface binding to panitumumab.

Peptides alone could not have sufficient immunogenicity to induce the production of antibody [4–8]. Heat shock proteins (HSPs), function as chaperones that stabilize and deliver peptides, are known as effective adjuvants to enhance peptide-specific tumor immunity [20, 21]. Vaccination with HSPs gp96, hsp90, and hsc70 derived from autologous cancer elicits specific immunity to tumors, protects mice from tumor challenge, delays cancer progression, and prolongs life-span [21]. Heat shock cognate protein 70 (hsc70) binding to synthetic peptides has been reported to elicit peptide-specific cytotoxic T lymphocytes (CTL) [22, 23]. Undono et al. identified that vaccination of murine hsc70 fused to CTL epitopes contributed to the priming of CD8^+^ T cells [22]. Moreover, hsc70 could serve as a connection between routes of antigen delivery, which may influence MHC II eptitope selection [23]. Therefore, in our study, to enhance the efficiency of peptide vaccine, mice were immunized with EGFR mimotope P19 or P26 fused to the C-terminus of mouse hsc70. Our results showed that Hsc70-P19 and Hsc70-P26 fusion protein could effectively elicit antibodies against the peptides, as well as the epitope of EGFR.

Many previous studies have reported the generation of peptide vaccines for induction of antibodies against EGFR [4–8]. On one hand, peptide vaccines were putative B-cell epitopes designed according to EGFR sequence. It has been reported that the peptides derived from separate sequences of EGFR could prevent tumorigenesis driven by EGFR or mutant EGFR [4, 8]. Moreover, rindopepimut (PEPvIII-KLH; CDX-110; Celldex Therapeutics, Phillipsburg, NJ), a 14-mer peptide which was derived from mutated EGFR variant III (EGFRvIII), could elicit both humoral and cellular immune responses. Phase II trial for GMB had verified the efficacy and safety of rindopepimut [9, 10]. Studies demonstrated that not only the EGFR B cell epitopes consisted of residues from EGFR contacting sites with EGF, but also the combination treatments of peptide vaccines exhibited significant anti-tumor effects *in vitro* or *in vivo* [4]. On the other hand, peptide vaccines could be identified as mimotopes by screening phage display peptide library targeting EGFR-specific antibodies [5–7]. Although no sequence homology was found between the mimotopes and EGFR, the peptide epitopes could mimic the conformational structure of EGFR, and induce antibodies against EGFR [5–7]. Interestingly, even screening the identical 7-mer phage library with cetuximab, completely different mimotope peptides could be isolated [6, 24]. Likewise, although mimitopes for panitumumab have been isolated from 12-mer phage peptide library by Voigt et al [25], here, we obtained different mimotopes, P19 and P26, from the same phage peptide library. These mimotopes were able to prime the active immunity against EGFR. The anti-mimotope cellular immune response possibly contains T helper cells, which could contribute to the production of specific antibody. Additionally, peptides are relatively nontoxic, more stable, low cost, easily penetrate tissue barriers, and having the potential to overcome resistance to panitumumab, which make them promising candidates for the development of most therapeutic agents [4]. However, there is still a long way to go before it could be applied in the clinic.

However, there are some limitations of this study. According to the 3D model for EGFR-panitumumab complex, the residues (Q384[EGFR], S418[EGFR], Q408[EGFR], H409[EGFR], I467[EGFR], S468[EGFR]), which played critical roles in EGFR-panitumumab interaction, are inconsistent with previous studies [25]. Therefore, further studies are required to confirm the roles of these residues,

In conclusion, peptide mimotope P19 and P26 selected with panitumumab by phage library screening are capable of mimicking the conformational structure of EGFR-panitumumab binding sites, and inducing both humoral and cellular immune responses against EGFR. These results raised the possibility that P19 and P26 could serve as candidate vaccines for active immunotherapy against EGFR-positive cancers.

## MATERIALS AND METHODS

### Phage library screening

The Ph.D.™-12 Phage Display Peptide Library Kit was purchased from New England Biolabs (NEB). Three consecutive rounds on panitumumab were performed according to the manufacturer's instructions and previous studies [26]. In each round of selection, to remove unspecific phages, 1.5×10^11^ independent phage clones were preabsorbed by human IgG (Beijing Zhongshan Golden Bridge Biotechnology Co Ltd, China) immobilized on rProtein A sepharose beads (GE Healthcare), followed by positive selection with panitumumab counterpart (Amgen Inc. US). Phages binding with panitumumab-protein A complex directly infected *E.coli* ER2738 for titer determination and amplification. The amplified phages were purified by precipitation with 20% PEG8000 and 2.5M NaCl for the next round. Three rounds of selection were performed.

### Phage ELISA and DNA sequencing

After three selection rounds, individual phage clones were amplified and determined for the binding to panitumumab by phage enzyme-linked immunosorbent assay (ELISA). ELISA plates were coated with 2μg/mL of panitumumab or control human IgG in 0.1M NaHCO_3_ (pH9.5), and then blocked with 5% skim milk. Bound phages were detected with an anti-M13 HRP-conjugated monoclonal antibody (GE Healthcare). A random single phage clone from original phage library was used as negative control. The reaction was developed with o-phenylenediamine (Beijing BioDee Biothechnology Co. Ltd, China) as substrate. OD_490_ was measured by using a microplate reader (Bio-Rad model 550). The positive clones were sequenced by Sangon Biotech Co. Ltd (Beijing, China).

### Synthesis of peptides

The peptides DTDWVRMRDSAR (P19), VPGWSQAFMALA (P26), and negative control peptide derived from a random single phage clone in original phage display peptide library, AHVAQHVIRTEA, were synthesized by Sangon Biotech Co. Ltd (Beijing, China). The purity of the peptides was > 98%, as assessed by HPLC.

### Expression and purification of recombinant GST peptide and His-Hsc70 peptide fusion proteins

The oligonucleotides encoding the peptides derived from positive (P19, P26) or control phage clones were synthesized by Sangon Biotech Co. Ltd (Beijing, China) and ligated into the pGEX-4T-1 and pET28a containing hsc70 coding sequence. The sequences of the oligonucleotide are listed in Supplementary Table 1. GST and His-Hsc70 fusion proteins were expressed in *E.coli* BL21 (Beijing ComWin Biotech Co Ltd, China), and purified with glutathione sepharose 4B beads (GE Healthcare, US) and Ni-NTA Agarose (QIAGEN), respectively, according to the manufacturer's instructions.

### Co-immunoprecipitation (co-IP) and GST pull-down assay

Co-IP and GST pull-down were performed as described previously [27]. Briefly, *E.coli* cell lysates containing GST fusion protein were prepared in lysis buffer (1% Triton X-100, 1 mmol/L PMSF, and 1 mg/mL lysozyme in PBS) followed by sonication. After centrifugation, supernatants were incubated with 2μg panitumumab plus 10μl rProtein A sepharose bead (GE healthcare), or glutathione sepharose 4B beads (GE Healthcare) for 4 h at 4°C, for co-IP and GST pull-down, respectively. After incubation, beads were washed with PBS three times. Bound proteins were subjected to 12% SDS-PAGE and subsequently immunoblotted with anti-GST and anti-human IgG antibodies (Beijing Zhongshan Golden Bridge Biotechnology Co Ltd, China).

### Competition ELISA

To determine the role of peptides on the inhibition of panitumumab binding to EGFR, 96-well plates were coated with 0.1μg/mL recombinant human EGF receptor (rhEGFR, Sino Biological Inc. Beijing, China), and then added the mixture of 0.2μg/mL panitumumab and dilutions of P19, P26, or control peptide (0, 2, 5, 10, 50, 100μg/mL). Bound panitumumab was detected by standard ELISA as described above. The inhibition was calculated by the formula: (OD_panitumumab_ - OD_panitumumab with peptide_) ×100%/OD_panitumumab_. The experiment was repeated three times.

### Cell lines and cell culture

Human cell lines A431, MCF7, and SW480 were purchased from American Type Culture Collection (ATCC, Manassas, VA). All cell lines were cultured in DMEM medium (Gibco). All the media were supplemented with 10% fetal bovine serum (FBS, PAA) and antibiotics (100 units/mL penicillin and 100 μg/mL streptomycin) at 37°C in a humidified incubator with 5% CO_2_.

### MTT proliferation assay

The proliferation assay was performed as previously described [26]. Briefly, the human epidermoid carcinoma A431 cells or human breast cancer MCF7 cells (1×10^4^ cells/well), with high and low expression levels of EGFR, respectively, were incubated overnight. On the following day, different concentrations of peptides (0, 2, 5, 10, 50, 100μg/mL) were added in 0.5% FBS media with 0.2μg/mL panitumumab or human IgG, and then incubated for 72h. All treatments were performed in triplicate. Numbers of live cells were determined using the MTT reagent by reading at 570 nm. The proliferation inhibition rate was calculated by the formula: (OD_human IgG_ – OD_panitumumab+peptide_) ×100%/OD_human IgG_.

To determine the effects of antibodies induced by Hsc70 fusion protein, MTT assay was also performed. A431, SW480, and MCF7 cells were plated in 96-well microtiter plate. On the following day, Antibodies isolated from immunized mice were added at increasing concentrations, and incubation was continued for 72h. The OD was detected as described above.

### Colony formation assay

Cells were seeded into 24-well plates (0.4 × 10^4^ cells/well) and cultured in the presence of antibodies with or without peptides as indicated. Cells were fixed, stained, and photographed after 10 days. More details are described [28]. All relevant assays were performed independently at least three times.

### Mice immunization and antibody purification

BALB/c mice 5 to 6 week old were used for immunization. Groups (n=5 per group) of mice were immunized subcutaneously with 50 μg of Hsc70-P19, Hsc70-P26, or Hsc70-control fusion protein emulsified in complete Freund's adjuvant at the first time. Mice were boosted 3 times with the fusion protein emulsified in incomplete Freund's adjuvant at 3-week intervals [29, 30]. Blood were taken from the tail vein on day 0 and on day 7 after every immunization. Serum was pooled from each group of mice by mixing equal volumes of fourth immune serum of each mouse. And then the sera were used for IgG purification by using rProtein A sepharose beads (GE Healthcare). The purified IgG were used for further ADCC, CDC, and cell proliferation assays.

To test the antibody responses against peptides and EGFR in the immunized mice, ELISA was performed as previously described [26]. Briefly, 96-well plates were coated with GST fusion protein or rhEGFR, and then the dilutions of serum from immunized mouse were added. The plate coated with GST was used as negative control. Bound antibodies were detected with HRP-conjugated goat anti-mouse IgG (Beijing Zhongshan Golden Bridge Biotechnology Co Ltd, China). The reaction was developed with o-phenylenediamine as substrate. OD_490_ was measured by using a microplate reader.

### Western blot

The response of immunized serum with EGFR protein was also determined by western blot. One μg of rhEGFR were electrophoresed followed by electrotransferring onto a nitrocellulose membrane. The proteins were probed with immunized serum. Anti-EGFR antibody (Cat#: 2239. 1:1000; Cell Signaling Technology) was used as positive control. And then the filters were incubated with HRP-conjugated secondary antibody. The signal was detected by the ECL western blot detection kit (Pierce).

To determine the effects of purified antibodies on EGFR-mediated signaling, cell lysates of A431 cells, pretreated with purified antibodies from immunized mice, panitumumab, or human IgG for 72h, were obtained in RIPA buffer. Total protein (100 μg) were subjected to 8% SDS-PAGE and transferred to nitrocellulose membrane. The expression of proteins was detected using primary antibody against pAKT (Cat#: 9271; 1:1000; Cell Signaling Technology), AKT (Cat#: 9272; 1:1000; Cell Signaling Technology), EGFR, and β-actin (Cat#: A1978; 1:2000; Sigma-Aldrich). The signal was detected as described above.

### Antibody-dependent cellular cytotoxicity (ADCC) assay

ADCC potentials of the antibodies induced by P19 or P26 were measured using the CytoTox 96 Non-Radioactive Cytotoxicity Assay (Promega) according to the manufacturer's instructions. Human peripheral blood mononuclear cells (PBMC) were used as effector cells [5, 31]. The target cell lines A431 or MCF7 were seeded in 96-well plates at a density of 1×10^4^ cells/well, and purified IgGs from immunized mice were added at 10μg/mL. After incubation at 37°C for 1h, effector cells were added and the cocultures were incubated for another 4h. The E:T ratio was 1:1, 10:1, 100:1. Cytotoxicity was calculated as follows: percentage of lysis (%) = (experimental – effector spontaneous – target spontaneous) ×100%/(target maximum– target spontaneous) [5, 31].

### Complement-dependent cytotoxicity (CDC) assay

For CDC assay, A431 or MCF7 cells (1×10^4^ cells/well) were cultured with 10μg/mL of purified antibodies from immunized mice for 4 h at 37°C, with 20% human serum co-incubation. The ability of antibodies to mediate CDC was measured by CytoTox 96 Non-Radioactive Cytotoxicity Assay (Promega), according to the manufacturer's instructions.

### Splenocyte proliferation assay

Spleen cells were isolated from 2 randomly selected BALB/c mice immunized four times with Hsc70-P19, Hsc70-P26 or Hsc70-control. 96-Well plates were coated with P19, P26 or control peptide at series of concentrations. The splenocytes (2×10^5^ cells/well) were cultured in the coated 96-well plates and incubated at 37°C for 72 h. Numbers of live cells were determined as described in “MTT proliferation assay”. The proliferation rate was calculated by the formula: (OD_P19/P26_ – OD_control_) ×100%/OD_control_.

### Cytokine assay by ELISA

The levels of IFN-γ and IL-4 in serum were measured using Mouse IFN-γ and IL-4 ELISA kits (Dakewei Inc, Beijing, China), according to the manufacturer's instructions. The absorbance was determined at 450 nm on a microplate reader (Bio-Rad model 550). Samples were tested in triplicates, and standard curve with mouse recombinant cytokine was made in each plate [32].

### BALB/c transplantable tumor model

BALB/c SCID mice at the age of 5-6 weeks were injected subcutaneously with 3 ×10^6^ A549 lung cancer cells [4]. At the same day, mice were treated i.p. with 300 μg of Hsc70-P19, Hsc70-P26, or Hsc70-control. The treatments were repeated once a week for 5 times. Tumor growth was measured once weekly using digital calipers. Tumor volume was calculated by the formula: 0.5 × L × W^2^ [33]. At the end of the experiment, the tumors were weighed.

### Modeling software

The sequences of the variable regions in heavy and light chain of panitumumab were submitted to the automated comparative protein modeling server SwissModel (http://swissmodel.expasy.org/) [14–16], to build up the three dimensional structures using the crystal structure of heavy chain of germline antibody PGT121-GL Fab (PDB: 4fqq) and light chain of RSV-neutralizing human antibody D25 (PDB: 4jha) as modeling templates, respectively.

The Docking program in the Insight II software package was used to generate the 3D models of panitumumab Fab by docking the two variable regions together. The model of EGFR-panitumumab complex was built using HEX 8.0.0 program [18]. In addition, The Docking program was also used to build the complex models of the peptide P19 and P26 with panitumumab, respectively. 3D figures were created with PyMOL (http://www.pymol.org/).

## SUPPLEMENTARY DATA


