# Supplementary material for: Induction of anti-EGFR immune response with mimotopes identified from a phage display peptide library by panitumumab
Source: Oncotarget. 2016 Sep 21;7(46):75293–306. doi: 10.18632/oncotarget.12167 (PMC5342741; doi:10.18632/oncotarget.12167)
Supplement: Supplementary file 1 [file oncotarget-07-75293-s001.pdf]

Induction of anti-EGFR immune response with mimotopes identified from a phage display peptide library by panitumumab

SUPPLEMENTARY DATA

SUPPLEMENTARY MATERIALS AND METHODS

Quantitative reverse transcription-PCR (qRT-PCR)

QRT-PCR was performed as described previously [1]. Briefly, total RNA from A431 and MCF7 cells were extracted using Trizol (Invitrogen, Carlsbad, CA, USA). The isolated total RNA was transcribed into cDNA using a reverse transcription kit (Promega, Madison, WI, USA) according to the manufacturer's instructions. Reverse transcription was followed by real time PCR with a SYBR Green PCR master mix (Toyobo Co. Ltd., Osaka, Japan). The specific primers used for EGFR and GAPDH mRNA were summarized in Table S1. Each sample was examined in triplicate.

Western blot

To determine the levels of EGFR in A431 and MCF7 cells, Western blot was performed as described previously [1]. Briefly, cell lysates of A431 or MCF7 cells were obtained in RIPA buffer. Total protein (100 μg) were subjected to 8% SDS-PAGE and transferred to nitrocellulose membrane. The expression of proteins was detected using primary antibody against EGFR and β-actin. The signal was detected by the ECL Western blot detection kit (Pierce).

REFERENCE

1. Wang Z, Jiang B, Chen L, Di J, Cui M, Liu M, Ma Y, Yang H, Xing J, Zhang C, Yao Z, Zhang N, Dong B, Ji J, Su X. GOLPH3 predicts survival of colorectal cancer patients treated with 5-fluorouracil-based adjuvant chemotherapy. J Transl Med. 2014; 12: 15.

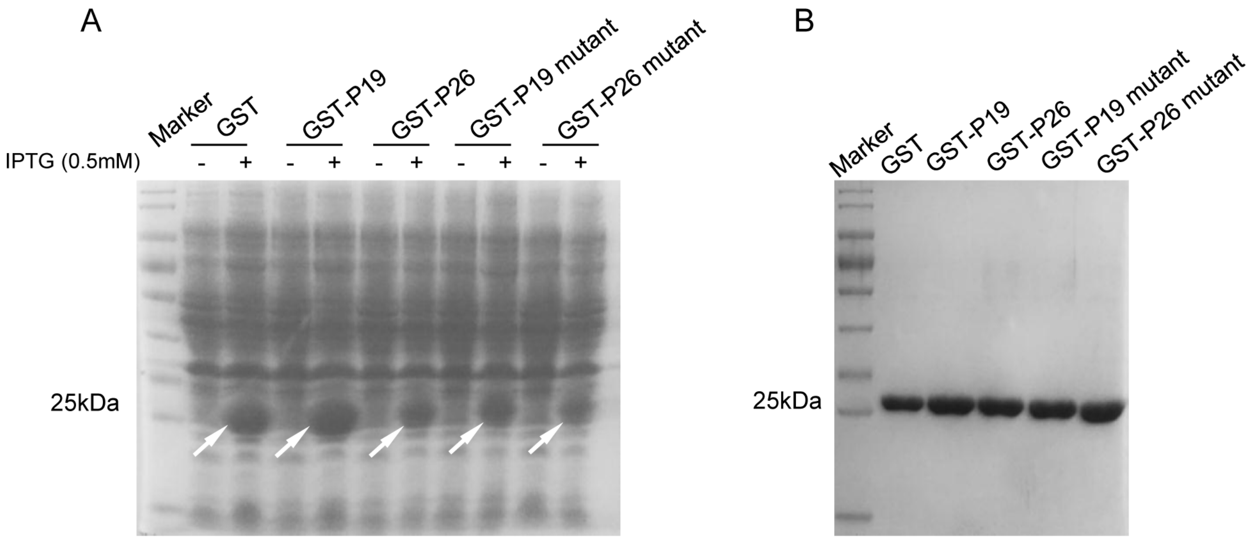

Supplementary Figure 1: Expression and purification of GST fusion protein. A. Expression of GST, GST-P19, GST-P26, GST-P19 mutant, and GST-P26 mutant in *E. coli* BL21 by IPTG induction. **B.** Purification of GST fusion protein. Proteins were separated by SDS-PAGE and visualized with coomassie blue staining. Arrows indicate the positions of expressed proteins.

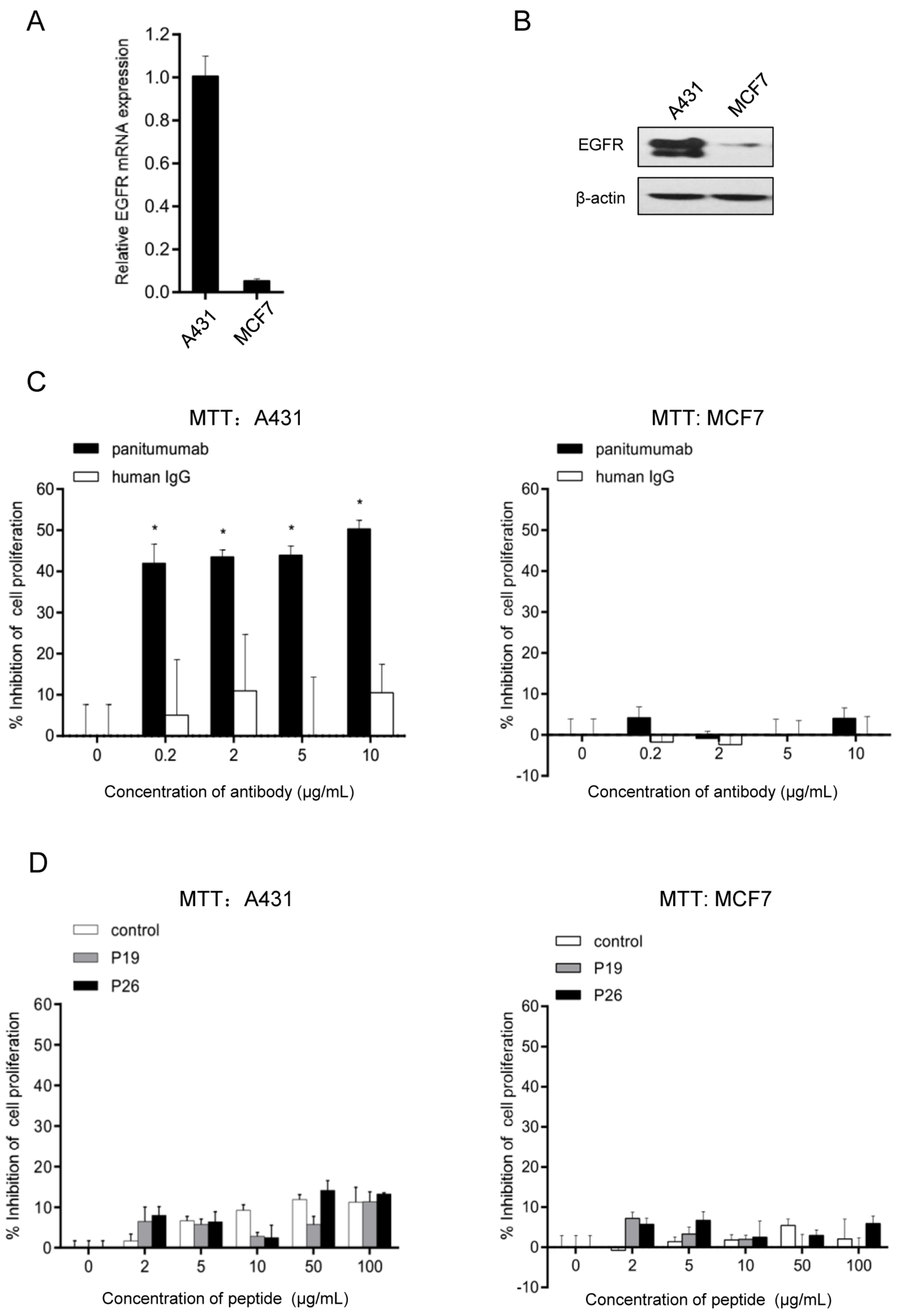

Supplementary Figure 2: The effects of panitumumab and peptides on the proliferation of tumor cells. Confirmation of EGFR levels in A431 and MCF7 cells by qRT-PCR **A.**, and Western blot **B.**, **C.** MTT assays of A431 (left panel) and MCF7 cells (right panel) treated with panitumumab or human IgG. **D.** MTT assays of A431 (left panel) and MCF7 cells (right panel) cultured with peptide P19, P26, and control. Data are shown as the mean (±SDs) of 3 independent experiments performed in triplicate. Statistically significant differences are indicated. *, $P < 0.05$.

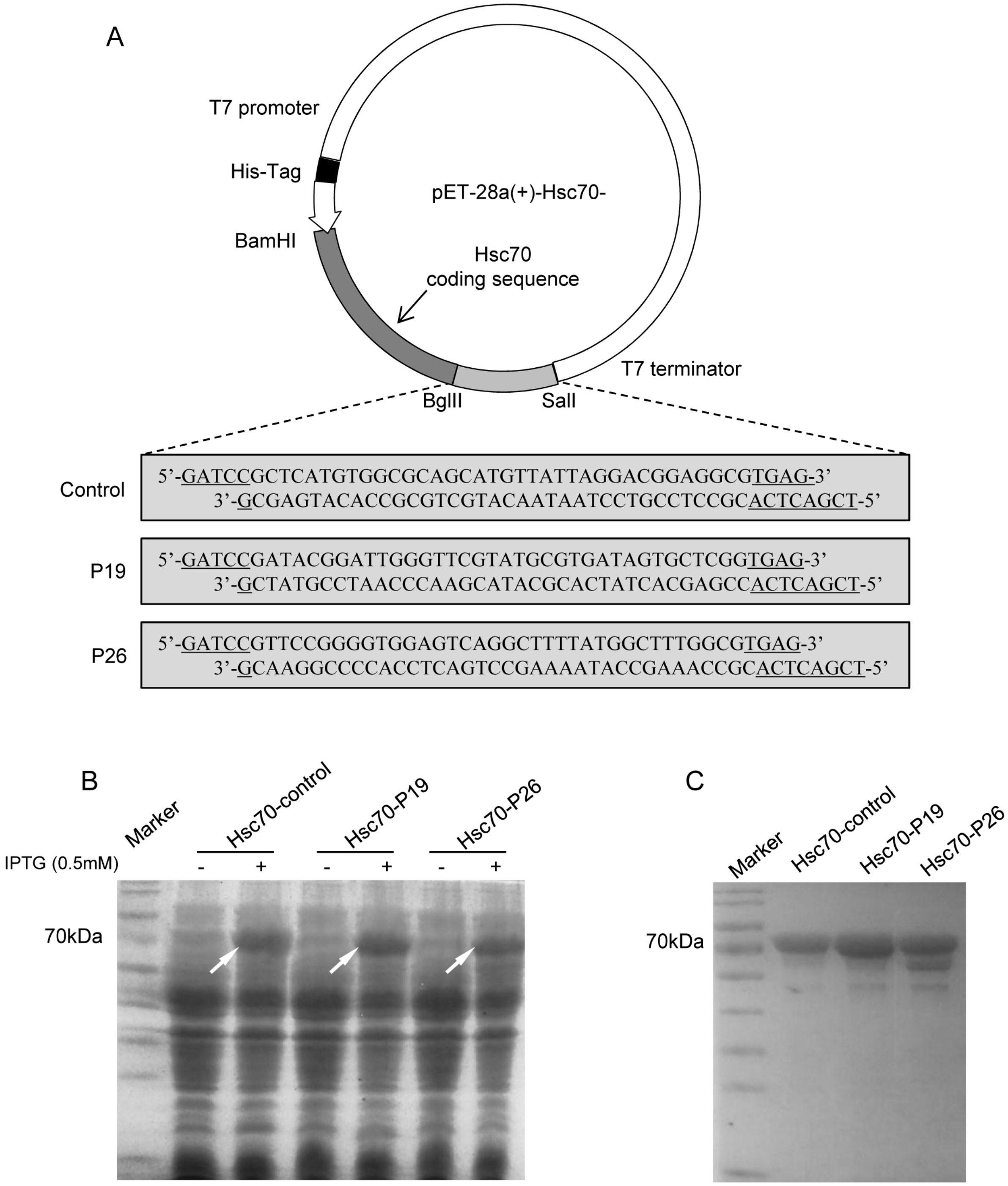

Supplementary Figure 3: Expression and purification of Hsc70 fusion protein. A. Diagram of the construction of plasmids encoding Hsc70-peptide fusion genes. Sequences encoding peptides were fused to the C-terminus of the hsc70 gene. **B.** Expression of Hsc70-control, Hsc70-P19, and Hsc70-P26 in *E. coli* BL21 by IPTG induction. **C.** Purification of Hsc70 fusion protein. Proteins were separated by SDS-PAGE and visualized with coomassie blue staining. Arrows indicate the positions of expressed proteins.

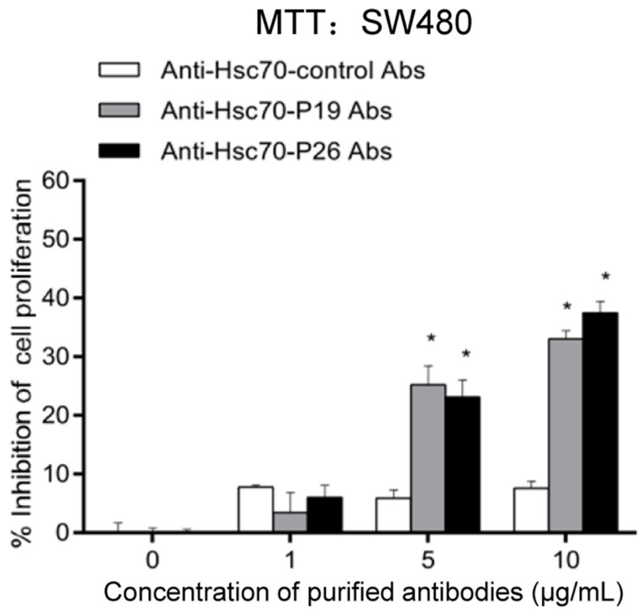

Supplementary Figure 4: MTT assay of SW480 cells treated with purified mimotope antibodies.

Supplementary Table 1: Sequences of primers

Primers	Sequences
**GST-P19**	
Forward primer	5′-GATCCGATACGGATTGGGTTCGTATGCGTGATAGTGCTCGGTGAGATATCG-3′
Reverse primer	5′-AATTCGATATCTCACCGAGCACTATCACGCATACGAACCCAATCCGTATCG-3′
**GST-P26**	
Forward primer	5′-GATCCGTTCCGGGGTGGAGTCAGGCTTTTATGGCTTTGGCGTGAGATATCG-3′
Reverse primer	5′-AATTCGATATCTCACGCCAAAGCCATAAAAGCCTGACTCCACCCCGGAACG-3′
**GST-P19 mutant**	
Forward primer	5′-GATCCGATACGGCAGCAGTTGCAATGGCAGATGCAGCTCGGTGAGATATCG-3′
Reverse primer	5′-AATTCGATATCTCACCGAGCTGCATCTGCCATTGCAACTGCTGCCGTATCG-3′
**GST-P26 mutant**	
Forward primer	5′-GATCCGTTCCGGGGGCAAGTGCAGCTGCAATGGCTGCAGCGTGAGATATCG-3′
Reverse primer	5′-AATTCGATATCTCACGCTGCAGCCATTGCAGCTGCACTTGCCCCCGGAACG-3′
**Hsc70-P19**	
Forward primer	5′-GATCCGATACGGATTGGGTTCGTATGCGTGATAGTGCTCGGTGAG-3′
Reverse primer	5′-TCGACTCACCGAGCACTATCACGCATACGAACCCAATCCGTATCG-3′
**Hsc70-P26**	
Forward primer	5′-GATCCGTTCCGGGGTGGAGTCAGGCTTTTATGGCTTTGGCGTGAG-3′
Reverse primer	5′-TCGACTCACGCCAAAGCCATAAAAGCCTGACTCCACCCCGGAACG-3′
**Hsc70-control**	
Forward primer	5′-GATCCGCTCATGTGGCGCAGCATGTTATTAGGACGGAGGCGTGAG-3′
Reverse primer	5′-TCGACTCACGCCTCCGTCCTAATAACATGCTGCGCCACATGAGCG-3′
**EGFR**	
Forward primer	5′-AGCTTCTTGCAGCGATACAGCTCAGAC-3
Reverse primer	5′-TGGGAACGGACTGGTTTATGTATTCAGG-3′
**GAPDH**	
Forward primer	5′-GGAGTCCACTGGCGTCTTCA-3′
Reverse primer	5′-GGGGTGCTAAGCAGTTGGTG-3′
